# Optical Sensing of Tissue Freezing Depth by Sapphire Cryo-Applicator and Steady-State Diffuse Reflectance Analysis

**DOI:** 10.3390/s24113655

**Published:** 2024-06-05

**Authors:** Arsen K. Zotov, Aleksandr V. Pushkarev, Anna I. Alekseeva, Kirill I. Zaytsev, Sergey S. Ryabikin, Dmitry I. Tsiganov, Dmitriy A. Zhidkov, Ivan A. Burkov, Vladimir N. Kurlov, Irina N. Dolganova

**Affiliations:** 1 Osipyan Institute of Solid State Physics of the Russian Academy of Sciences, Chernogolovka 142432, Russia; akzotov@issp.ac.ru (A.K.Z.);; 2 Prokhorov General Physics Institute of the Russian Academy of Sciences, Moscow 119991, Russia; 3 Bauman Moscow State Technical University, Moscow 105005, Russia; 4 Federal State Budgetary Educational Institution of Further Professional Education “Russian Medical Academy of Continuous Professional Education”, Ministry of Healthcare of the Russian Federation, Moscow 125993, Russia; 5 Avtsyn Research Institute of Human Morphology, FSBSI “Petrovsky National Research Centre of Surgery”, Moscow 117418, Russia

**Keywords:** cryosurgery, tissue freezing, sapphire, crystal growth, diffuse reflectance analysis, optical sensing

## Abstract

This work describes a sapphire cryo-applicator with the ability to sense tissue freezing depth during cryosurgery by illumination of tissue and analyzing diffuse optical signals in a steady-state regime. The applicator was manufactured by the crystal growth technique and has several spatially resolved internal channels for accommodating optical fibers. The method of reconstructing freezing depth proposed in this work requires one illumination and two detection channels. The analysis of the detected intensities yields the estimation of the time evolution of the effective attenuation coefficient, which is compared with the theoretically calculated values obtained for a number of combinations of tissue parameters. The experimental test of the proposed applicator and approach for freezing depth reconstruction was performed using gelatin-based tissue phantom and rat liver tissue in vivo. It revealed the ability to estimate depth up to 8 mm. The in vivo study confirmed the feasibility of the applicator to sense the freezing depth of living tissues despite the possible diversity of their optical parameters. The results justify the potential of the described design of a sapphire instrument for cryosurgery.

## 1. Introduction

Cryosurgery and cryotherapy are associated with the application of low temperatures to living tissues and cold-induced effects, such as necrosis, osmotic injury, tissue hypoxia, cell apoptosis, as well as hemostatic and immunological effects [1,2,3,4]. Cryosurgery finds its applications in oncology, dermatology, gynecology, urology and other fields for the surface or interstitial non-surgical removal of various lesions and neoplasms [5,6,7,8,9,10,11]. The advantages of cryosurgery also include minimal invasiveness, relative painlessness and short recovery time for patients [12,13].

Since cryosurgery is aimed at cold-induced tissue ablation during freezing, it is connected with the risk of damaging the surrounding healthy tissues as well as the incomplete freezing of the targeted tissue volume [14]. Several approaches have been applied to addressing this problem. The first one deals with the accurate planning of the procedure, numerical simulations and preliminary estimations [15,16,17,18]. The evident drawback of this approach is the absence of real-time monitoring of tissue freezing. Therefore, it is desirable to use other techniques for monitoring, such as ultrasound (US) [19,20] and magnetic resonance imaging (MRI) [21,22,23], computed tomography (CT) [24,25] and temperature measurements [26]. Visualization by US, CT and MRI requires additional sensors or even expensive systems that can not be combined with the cryosurgical instrument. Moreover, MRI needs non-magnetic cryosurgical equipment and CT images can suffer from the artifacts caused by the usage of common metal cryo-applicators, which hinder the visualization of the so-called ice ball formation in tissue. Temperature measurements enable estimation of tissue condition only at several points or on its surface. Among other approaches, we should also mention the application of cryo-protective agents, which help to manage, to some extent, the freezing volume and protect, for example, adjacent nerves [27].

To solve the stated problem of monitoring the cryosurgical process, it is attractive to use optical sensors and methods, such as analysis of the opto-acoustic signal [28,29,30], terahertz monitoring [31], and diffuse reflectance analysis [32,33]. The latter can be implemented with optical fibers and, thus, be considered as a part of a cryosurgical instrument. We have recently suggested the concept of a sapphire applicator for cryosurgery with the ability to detect tissue freezing depth using the analysis of spatially resolved diffuse reflected intensities [34]. Sapphire provides biocompatibility and chemical inertness together with high performance of tissue freezing, since it features high thermal conductivity at cryogenic temperatures [35,36]. It has been demonstrated that a submerged sapphire applicator enables faster tissue freezing and lower temperatures in comparison with metal ones [37]. Moreover, the application of crystal growth techniques [38,39,40] allows for the manufacturing of sapphire shaped crystals with low amounts of surface and volume defects, which, together with the high transparency of sapphire in visible and near-infrared (NIR) ranges, gives the opportunity to deliver light to tissues through the contact part of the applicator. Thus, optical methods of tissue monitoring during cryosurgery can be implemented directly in the sapphire instrument.

The principle of freezing-depth sensing by a sapphire applicator was described in our previous work [34]. Using spatially resolved optical fibers accommodated inside the crystal and connected to the light source and the detector, the diffuse reflected intensities are analyzed during the formation of an ice ball inside the tissue. The reconstruction of the ice ball height, i.e., the freezing depth, is implemented based on the principles of light diffusion in a turbid medium and frequency modulation of the light source [41,42,43,44,45]. However, this applicator needs rather complicated optical equipment and analysis of signals in the frequency domain. Therefore, in the present work, we propose another implementation of the mentioned optical sensing approach, and we studied the possibility of using steady-state analysis of diffuse reflected intensities for freezing-depth reconstruction. We used a multi-channeled sapphire applicator manufactured by the crystal growth technique. One channel serves for the illumination of the sample, while two other channels serve for detection of the diffuse signal. The experimental approbation of the suggested approach was performed with a tissue-mimicking phantom and in vivo rat liver tissues. The results show the advantages and drawbacks of this applicator and approach. Thus, this work demonstrates the feasibility of the optical sensing of tissue freezing by means of a sapphire cryo-applicator and the analysis of spatially resolved optical responses in steady-state regimes.

## 2. Materials and Methods

### 2.1. Multi-Channeled Sapphire Applicator

**Figure 1:**
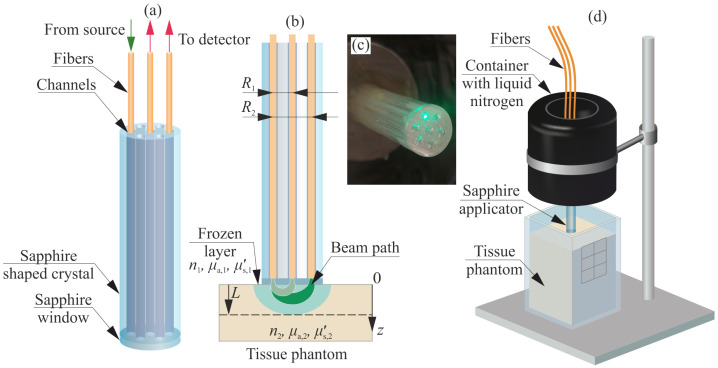
A sapphire applicator: (**a**) Schematic of a multi-channeled 12 mm diameter applicator with optical fibers accommodated inside three channels. (**b**) Schematic of beam path of the detected optical diffuse signals, $R_{1}=4$ mm, $R_{2}=8$ mm. (**c**) A photo of the sapphire applicator. (**d**) The experiment of an ice ball formation in a tissue phantom by the sapphire applicator.

A sapphire applicator with an outer diameter of 12 mm and a length of 149 mm is shown in Figure 1. It was manufactured by the edge-defined film-fed growth (EFG) technique [39] and has internal channels with a diameter of 2 mm for accommodating optical fibers inside the applicator. EFG and related techniques help to obtain shaped crystals with as-grown quality of channel surface without additional mechanical processing. The manufacturing of a crystal by EFG technique requires a growth chamber with a high-purity Ar atmosphere as an ambient under a pressure of 1.1–1.3 atm, a molybdenum crucible with a 22 kHz induction-heated graphite susceptor and a molybdenum die with capillary channels. The die determines the crystal’s cross-section. To maintain the growth process, the applied set-up is equipped by an in-house automated weight control system. The considered crystal shape and the channel size, which is larger than the common fiber diameter, allow for avoiding the appearance of crystal defects and gas inclusions during the growth process. On the other hand, the decreasing of the channel diameter complicates the efforts for preventing the channel collapse by maintaining the heating zone parameters.

To perform optical monitoring of the sample by the sapphire applicator, optical fibers (with diameter of 200 μm, NA =0.22) are accommodated and fixed by epoxy adhesive inside the three channels of the crystal. The first fiber is used for tissue illumination; it is connected to a laser diode (M530F2, Thorlabs, Inc., Newton, NJ, USA) with a central wavelength of 530 nm and minimal output power of 6.8 mW. For this study, a green light source was chosen for implementing the feasibility test of sensing the tissue phantom, which features low absorption, and approving the tested applicator configuration by using several tissues in vivo. However, for further studies, tissue absorption properties should be taken into account and a red/NIR light source is preferable. Nevertheless, the applied source wavelength does not change the method of reconstructing tissue freezing depth, described below. The other two fibers are used for the detection of the diffused optical response; each one being connected to a separate spectrometer (CCS100/M, Thorlabs, Inc., Newton, NJ, USA). The signals are registered within a 3 s period during the application. To protect the fibers from direct contact with the tissue, a 2 mm thick sapphire window is bounded to the bottom end of the applicator by an adhesive layer with a thickness of 200 μm. The remaining channels are assumed to be used for possible additional monitoring or exposure, but they are not involved in the present implementation of optical monitoring. The sapphire window also helps to homogenize the cooling profile over the application time, which is slightly changed by the presence of the channels.

The sapphire applicator is mounted in a thermally insulated tank with an outer diameter of 100 mm (see Figure 1d). The thickness of the thermal insulation is 15 mm. In the bottom, the tank has a threaded fastener for fixation of the applicator. In cases of using liquid samples, they are stored in the container with 90×90×110 mm dimensions.

### 2.2. Reconstruction of a Freezing Depth

During the sample freezing, diffuse reflected intensity I(R,L) is recorded at each moment *t*; here, *R* is the distance between the illumination and detection channels (or the source–detector separation) and *L* is the freezing depth (see Figure 1b). Using the applicator described above, the distance *R* is equal to 4 and 8 mm for two detection channels. The signals are then smoothed by moving average filtering.

For a semi-infinite turbid medium, according to the diffuse approximation of the radiation transfer equation [46,47], the diffuse reflectance can be estimated as

(1) $$I\left(R\right)=\frac{C_{1}}{R^{m}}\exp\left(-C_{2}R\right),$$

where $C_{1}$ and $C_{2}$ are empirically determined coefficients depending on optical parameters of the turbid medium and parameter *m* depends on the distance *R*. For the considered design of the applicator, we set m=1.5.

An effective attenuation coefficient $\mu_{\mathrm{eff}}$ of the medium describes its absorption and scattering properties:

(2) $$\mu_{\mathrm{eff}}={\left[3\mu_{a}\left(3\mu_{a}+\mu_{s}^{\prime }\right)\right]}^{1/2},$$

where $\mu_{a}$ and $\mu_{s}^{\prime }$ are the absorption and reduced scattering coefficients, respectively. From Equation (1), $\mu_{\mathrm{eff}}$ can be estimated as $\mu_{\mathrm{eff}}≃C_{2}$. Consequently, using two detection channels and measuring $I(R_{1})$ and $I(R_{2})$, the effective attenuation coefficient is calculated from Equation (1):

(3) $$\mu_{\mathrm{eff}}≃\frac{\ln\left[I\left(R_{1}\right)R_{1}^{m}\right]-\ln[I\left(R_{2}\right)R_{2}^{m}]}{R_{2}-R_{1}}.$$

In order to evaluate correctly $\mu_{\mathrm{eff}}$ from the measured signals, the applicator needs calibration using a sample with known optical parameters. The calibration should be performed with a preliminary cooled applicator, and only the initial values of $I_{\mathrm{calib}}\left(R,L=0\right)$—which are obtained before the freezing layer begins to appear—should be applied in the calculations. We compare the slope $\ln\left(IR^{m}\right)$ obtained for the actual measurement of the calibration phantom with the slope of a fit line $\left[\ln C_{1}-C_{2}R\right]$, where $C_{2}$ is estimated from Equation (2). As a result, the required corrections for the measurements are determined.

For reconstruction of freezing depth, we assume the plane-parallel structure of the tissue-mimicking phantom, with an upper layer of frozen medium of thickness *L* and a bottom semi-infinite layer of unfrozen medium. Theoretically, the diffuse reflectivity from the two-layered turbid medium is described by the following formulas, assuming the extrapolated boundary condition [44]:

(4) $$I_{\mathrm{th}}\left(R,L\right)=D_{1}\frac{\partial }{\partial z}\hat{\Phi }{\left(R,z,L\right)|}_{z=0}$$

(5) $$\hat{\Phi }\left(R,z,L\right)=\frac{1}{\pi a^{\prime 2}}\sum_{n=1}^{\infty }G\left(s_{n},z,L\right)J_{0}\left(s_{n}R\right)J_{1}^{-2}\left(a^{\prime }s_{n}\right),$$

(6) $$\begin{array}{cc} G(s_{n},z,L)= & \frac{\exp\left(-\alpha_{1}|z-z_{0}|\right)-\exp[-\alpha_{1}\left(z+z_{0}+2z_{b}\right)]}{2D_{1}\alpha_{1}}\\ \; & +\frac{\sinh\left[\alpha_{1}\left(z_{0}+z_{b}\right)\right]\sinh\left[\alpha_{1}\left(z+z_{b}\right)\right]}{D_{1}\alpha_{1}\exp\left[\alpha_{1}\left(L_{1}+z_{b}\right)\right]}\\ \; & \times \frac{D_{1}\alpha_{1}-D_{2}\alpha_{2}}{D_{1}\alpha_{1}\cosh\left[\alpha_{1}\left(L_{1}-z_{b}\right)\right]+D_{2}\alpha_{2}\sinh\left[\alpha_{1}\left(L_{1}-z_{b}\right)\right]}, \end{array}$$

(7) $$\alpha_{k}=\sqrt{\mu_{a,k}/D_{k}+s_{n}^{2}},$$

where $\hat{\Phi }\left(R,z,L\right)$ is the photon fluence in the upper layer at an arbitrary position *R* relative to the source position, *z* is the sample depth direction, *L* is the thickness of the upper frozen layer, $D_{k}=1/\left(3\mu_{s,k}^{\prime }\right)$ is the diffusion coefficient of the *k*-layer (1, 2 stand for upper frozen and bottom unfrozen layers, respectively), $\mu_{s,k}^{\prime }$ and $\mu_{a,k}$ are the reduced scattering and absorption coefficients, respectively, of the *k*-layer, $G(s_{n},z,L)$ is the Green’s function, $J_{p}$ is the Bessel function of the first kind and order *p*, $a^{\prime }=a+z_{b}$, *a* is the characteristic radius of the sample cylinder, $z_{b}$ describes the position of the extrapolated boundary and depends on the refractive indices and scattering coefficients of two layers [41], $z_{0}=1/\mu_{s,1}^{\prime }$ and $s_{n}$ is the positive root of the equation $J_{0}\left(a^{\prime }s_{n}\right)=0$. Thus, after numerical calculation of the photon fluence in the upper layer, finding its first derivative on the sample surface, the diffuse reflectivity $I_{\mathrm{th}}\left(R,L\right)$ can be obtained for $R_{1}$ and $R_{2}$, which yields the theoretical estimation of the effective attenuation $\mu_{\mathrm{eff},\mathrm{th}}$ by means of Equation (3) for arbitrary thickness *L*. In these calculations, we assume that the sample optical parameters may vary in some ranges. Therefore, we obtain $I_{\mathrm{th}}\left(R,L\right)$ and $\mu_{\mathrm{eff},\mathrm{th}}=\mu_{\mathrm{eff},\mathrm{th}}\left(L,n_{1},n_{2},\mu_{a,1},\mu_{a,2},\mu_{s,1}^{\prime },\mu_{s,2}^{\prime }\right)$ for this set of parameters. The $\mu_{\mathrm{eff},\mathrm{th}}$ is then compared with $\mu_{\mathrm{eff},\mathrm{meas}}\left(t\right)$ obtained from the actually measured $I_{\mathrm{meas}}\left(R,t\right)$. Minimization of error at every particular moment *t* gives the freezing-depth dependence L(t):

(8) $$L\left(t\right)={\mathrm{argmin}}_{L}\left|\mu_{\mathrm{eff},\mathrm{meas}}\left(t\right)-\mu_{\mathrm{eff},\mathrm{th}}\left(L,n_{1},n_{2},\mu_{a,1},\mu_{a,2},\mu_{s,1}^{\prime },\mu_{s,2}^{\prime }\right)\right|.$$

The condition $L_{i+1}>L_{i}$ for the $t_{i+1}>t_{i}$ moment is applied.

## 3. Results

### 3.1. Calibration

**Figure 2:**
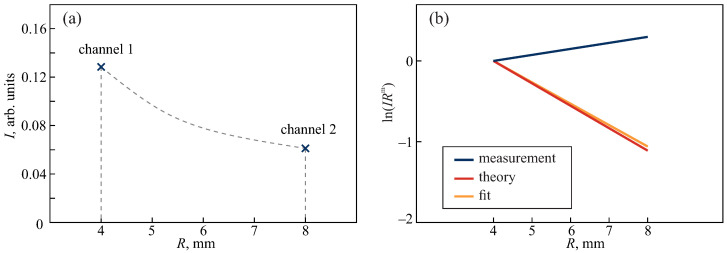
Calibration of the applicator: (**a**) The measured intensities at two channels with $R_{1}=4$ and $R_{2}=8$ mm. An aqueous 3% solution of Lipoplus 20 was used as a sample. (**b**) Comparison of the actually measured signal slope, its theoretical estimation and the fit line $\left[\ln C_{1}-C_{2}R\right]$, where $C_{2}≃\mu_{\mathrm{eff}}$ and $\mu_{\mathrm{eff}}$ were determined from Equation (2). All lines were shifted to zero at $R_{1}$ for simplicity.

For the calibration of the applicator, we used an aqueous 3% solution of lipid emulsion (Lipoplus 20, B. Brown, Melsungen, Germany). It has thermal [48,49] and optical [50,51,52] properties similar to biotissues. The phantom temperature was ambient (24 °C). The results of the calibration are shown in Figure 2. Here, panel (a) demonstrates the measured intensities. By means of Equations (1) and (2) we calculated the fitted slope (orange line in Figure 2b) and by means of Equations (4)–(7) we calculated $I_{\mathrm{th}}\left(R\right)$ for the calibration phantom, assuming that it consisted of a single unfrozen layer. In Figure 2b, we show the corresponding slope $\ln\left[I_{\mathrm{th}}\left(R\right)R^{m}\right]$. The measurement, fit and theoretical slopes were vertically shifted to zero at $R_{1}$. It is evident that $\ln\left[I_{\mathrm{th}}\left(R\right)R^{m}\right]$ almost matches the fit line, which justifies the algorithm for theoretical calculation. On the other hand, the noticed significant difference between the actually measured and the theoretical slopes can be explained by the uneven sensitivity of the spectrometers, fiber losses and possible reflections of the light beam in the sapphire window. The calibration of the probe helped to eliminate the measurement error caused by these reasons.

### 3.2. Sensing of Tissue Phantom Freezing

**Figure 3:**
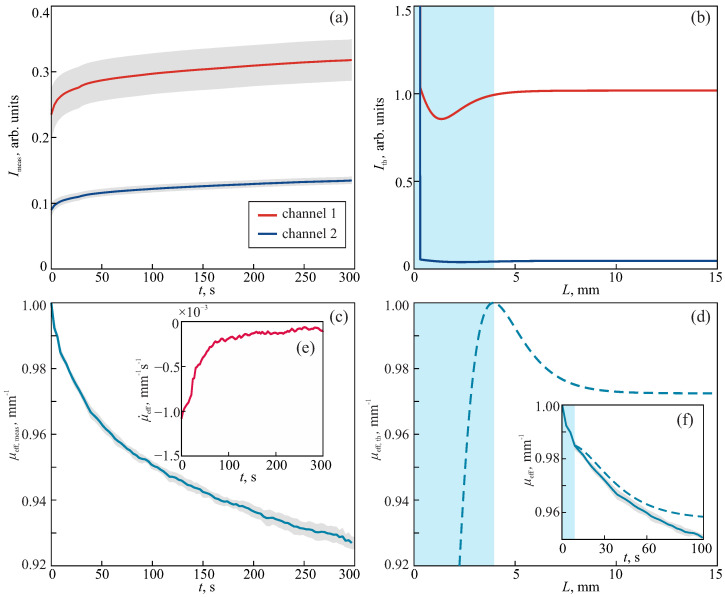
Signal analysis during the tissue phantom freezing: (**a**,**b**) Time and thickness evolution of normalized diffuse reflected intensities $I_{\mathrm{meas}}$ and $I_{\mathrm{th}}$, respectively, detected at two channels separated from the illumination channel by distance *R*; $I_{\mathrm{meas}}$ is shown after smoothing filtering. (**c**,**d**) Normalized effective attenuation coefficient obtained from the measured signals and calculated intensities, respectively. (**e**) The rate of change of the measured attenuation. (**f**) Comparison of $\mu_{\mathrm{meas}}$ and $\mu_{\mathrm{th}}$, assuming a certain time delay indicated by the blue region, aimed at *L* reconstruction by Equation (8). Gray regions denote measurement errors.

For the experimental approbation of the sapphire applicator, the tissue-mimicking phantom was applied. It contained gelatin with 95±1% moisture content and Lipoplus 20 of 1% volume concentration. The phantom was made directly in the sample container (Figure 1d), which eliminated the presence of air bubbles. Its initial temperature was ambient (24 °C). The preliminary cooled applicator touched the surface of the phantom to start the formation of an ice ball. During this process, the optical signals were stored. They are shown in Figure 3a. After freezing, the phantom was thawed at room temperature. The whole experiment was repeated three times.

For measuring the ice ball dimensions, the growth process was recorded by a digital camera (Sony a7 mark II with lens Art 24-70 f/2.8 FE). We should note that to visualize the ice ball growth, the phantom should be transparent in the visible range; therefore, it should not contain lipid emulsion for this measurement. Thus, the dimensions were recorded for a pure gelatin medium. We assumed that the ice ball formation was mainly governed by the gel properties; thus, the absence of emulsion caused negligible errors in measuring the ice ball size in the phantom.

Considering the dispersion of gelatin properties, as well as a slight sedimentation of lipid inclusions in warm gelatin during solidification, we set the variance of the phantom parameters, in order to calculate $I_{\mathrm{th}}\left(R,L\right)$. Since the scattering and absorption properties of the phantom were mostly determined by the lipid emulsion, which has optical properties similar to those of intralipid [53,54], we assumed the following parameters for the unfrozen layer: refractive index $n_{2}=\left[1.40–1.42\right]$, reduced scattering coefficient $\mu_{s,2}^{\prime }=\left[0.50–0.85\right]$ ${\mathrm{mm}}^{-1}$, absorption coefficient $\mu_{a,2}=\left[0.03–0.05\right]$ ${\mathrm{mm}}^{-1}$. These data were in accordance with the previously measured properties of such phantoms [52,53]. Then, we assumed that the frozen layer was characterized by the slight alteration of the refractive index $n_{1}=n_{2}-\Delta n$, Δn=[0.02–0.04], and that the absorption coefficient $\mu_{a,1}=\mu_{a,2}-\Delta \mu_{a}$, $\Delta \mu_{a}=\left[0.008–0.01\right]$ ${\mathrm{mm}}^{-1}$. At the same time, the reduced scattering coefficient increased as $\mu_{s,1}^{\prime }=\mu_{s,2}^{\prime }+\Delta \mu_{s}^{\prime }$, where $\Delta \mu_{s}^{\prime }=\left[0.5–1.0\right]$ ${\mathrm{mm}}^{-1}$. The introduced ranges for the properties of the frozen phantom were adjusted numerically. The expanding of these boundaries yielded the same results of depth reconstruction, but increased the computation time. Figure 3b demonstrates the intensity $I_{\mathrm{th}}\left(R,L\right)$ obtained for certain sample properties from the described ranges. In contrast to the behavior of $I_{\mathrm{meas}}$, we can notice uneven alteration of $I_{\mathrm{th}}$ at small freezing depth *L*. The obtained $I_{\mathrm{th}}$ decreases to a certain value before starting to increase, which is explained by the usage of extrapolated boundary conditions. While the measured intensities yielded the reduction of $\mu_{\mathrm{eff},\mathrm{meas}}$ during the ice ball formation (the normalized $\mu_{\mathrm{eff},\mathrm{meas}}$ is shown in Figure 3c), the reduction of $\mu_{\mathrm{eff},\mathrm{th}}$ began after a certain depth *L* (Figure 3d). Thus, we should begin to compare the measured and calculated effective attenuation coefficient and the reconstructed *L* by Equation (8), assuming some delay from the beginning of freezing (see Figure 3f). This delay is indicated by a blue region in panel (f), which corresponds to a certain depth range, shown by the same color in panels (b) and (d).

**Figure 4:**
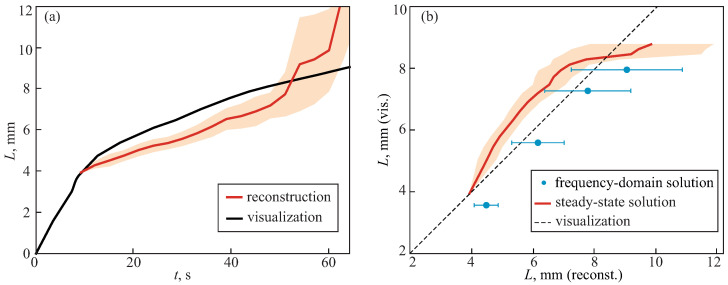
Reconstruction of freezing depth in tissue phantom performed by the sapphire cryo-applicator: (**a**) Time evolution of the reconstructed depth and the visualized ice ball height in gelatin phantom. (**b**) Comparison of the reconstructed and visualized freezing depth for frequency-domain solution (data from Ref. [34]) and steady-state solution. The region of error bars represent ±σ.

Figure 4 shows the reconstructed freezing depth and its comparison with the visualized ice ball height. The proposed approach ensured rather good agreement between two values within a time range from 10 to 50 s. The error of the depth estimation was <1.5 mm. It increased dramatically after 50 s and L=8 mm. This meant that the size and curvature of the ice ball did not allow for monitoring of the freezing depth by the applicator with the considered positions of the channels. To increase the upper limit of depth reconstruction, one should choose a larger distance between the illumination and detection channels along with the account of the ice ball curvature instead of using plane-parallel approximation, which will lie within the scope of our further studies. However, a large source-detector separation is appropriate only for superficial applicators. Otherwise, the illumination of tissue should be performed by a separate tool, which will complicate the overall instrumentation. At the same time, for this particular sample medium, reconstruction of the freezing depth is impossible for t<10 s, due to the reason described above.

In Figure 4b, the results of the performed depth sensing are compared with those obtained by the use of frequency-domain solution and four detection channels [34]. Despite applying only two detection channels and a steady-state solution, the errors of the two approaches are almost comparable, but slightly increased for the present applicator. Moreover, the range of freezing depth that can be reconstructed is the same.

We should note that possible errors of reconstruction can be caused by the freezing and thawing of the phantom, which contains lipid emulsion. In order to fix the optical parameters of the phantom medium during several experiments, the measurements were performed with the same phantom volume. Therefore, freezing and thawing of lipid emulsion may still have caused change in the phantom properties, similar to the changes of real tissue properties. Indirectly, this was confirmed by a rather high variance of the measured I(t) (Figure 3a). This fact leads to rather high variance of depth reconstruction and imposes the necessity of using a rather wide range of sample initial parameters for theoretical calculations. To overcome this problem, it is possible to use additional methods of measuring initial sample parameters, which would help to strength the results of reconstruction and reduce the calculation efforts.

### 3.3. Sensing of In Vivo Rat Liver Freezing

**Figure 5:**
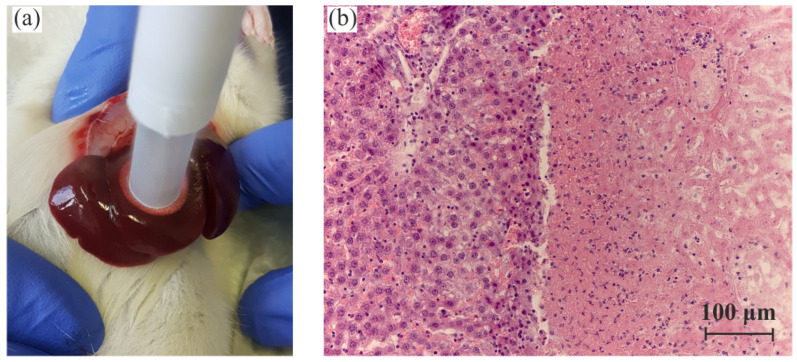
The freezing of rat liver by the sapphire cryo-applicator: (**a**) An image of the freezing process. (**b**) A representative H&E histological image of the liver tissue after cold-induced damage; the damaged tissue is shown on the right.

To continue the experimental approbation of the proposed sapphire cryo-applicator, we studied the cryodestruction of in vivo liver tissue. For this aim, four Wistar rats (females) weighing 280–380 g were used. The animals were placed under deep anesthesia using Zoletil and xylazine (Interchemie, Püünsi, Estonia)/propofol (Fresenius Kabi, Graz, Austria). After treating the wound surface with an antiseptic, an incision about 4–5 cm long was made to the right of the white midline of the abdomen in the hypochondrium. The skin and muscle wall were incised. Next, several lobes of the liver were isolated (raised to the surface) for immediate cryo-application (Figure 5a). The applicator was cooled down before the experiment. The application was carried out for 5–20 s, namely 5 s (sample 1), 15 s (samples 2 and 3) and 20 s (sample 4). Due to the rather small thickness of the liver lobes, longer exposure could induce total freezing. After thawing, the liver lobe was carefully removed back into the abdominal cavity and sutured with silk threads in layers.

The rats were removed from the experiment in the ${\mathrm{CO}}_{2}$ camera 24 h after the cryo-application. After measuring the dimensions of the damaged tissue area, liver specimens were taken for characterization to confirm the destruction of the tissue. They were fixed in formaldehyde solution (4% paraformaldehyde solution, pH =7.4) for 48 h, immersed in ethanol–xylene solutions and embedded in paraffin blocks. Then, sections with a thickness of 4–7 μm were made and were stained with hematoxylin and eosin (H&E). Histological images of the specimens, were obtained at magnification ×200. Figure 5b shows a representative H&E-stained image of the rat liver tissue after cold-induced damage. The border between healthy (on the left) and damaged (on the right) tissue regions was smooth; the damaged region was characterized by either moderate or severe vasodilation and edema. As one can notice, the damaged tissue may feature uneven consistency. Consequently, the parameters of the probed tissue region may change during the application, as the thickness of the frozen layer increases. In order to take this variation into account during obtaining $\mu_{\mathrm{eff},\mathrm{th}}$, we assumed that the equivalent homogeneous damaged tissue could be considered, which was characterized by the averaged parameters within the specific ranges.

**Figure 6:**
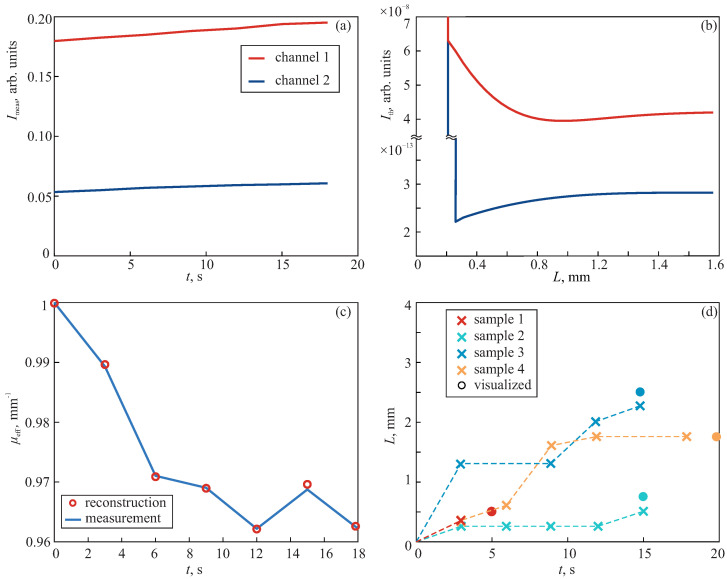
The results of monitoring the cryodestruction of rat liver tissue: (**a**,**b**) Time and thickness evolution of normalized diffuse reflected intensities $I_{\mathrm{meas}}$ and $I_{\mathrm{th}}$, respectively, detected at two channels separated from the illumination channel by distance *R*; $I_{\mathrm{meas}}$ is shown after smoothing filtering. (**c**) Normalized effective attenuation coefficient obtained from the measured signals and calculated intensities. Data are shown for sample 4. (**d**) Time evolution of the reconstructed freezing depth (crosses) compared with the visualized depth (circles) at the end of exposure.

Figure 6 demonstrates the results of this experiment. In Figure 6a, we show a representative time evolution of $I_{\mathrm{meas}}$ for one sample. However, the measured intensities varied significantly from sample to sample. This is explained by the different thicknesses of the liver lobes as well as by the variance of optical properties for different animals. As described in Section 2.2, from $I_{\mathrm{meas}}\left(R,t\right)$, we could estimate the effective attenuation coefficient (Figure 6c). Since the optical signals were measured within a period of 3 s, $\mu_{\mathrm{eff},\mathrm{meas}}\left(t\right)$ was characterized by only a few points.

To obtain $I_{\mathrm{th}}\left(R,L\right)$, the following tissue parameters were used. The unfrozen layer was characterized by $n_{2}=\left[1.37–1.40\right]$, $\mu_{s,2}^{\prime }=\left[2.70–2.8\right]$ ${\mathrm{mm}}^{-1}$ and $\mu_{a,2}=\left[1.00–1.15\right]$ ${\mathrm{mm}}^{-1}$, while for the frozen layer we assumed $n_{1}=n_{2}-\Delta n$, Δn=[0.02–0.1] $\mu_{s,1}^{\prime }=\mu_{s,2}^{\prime }+\Delta \mu_{s}^{\prime }$, $\Delta \mu_{s}^{\prime }=\left[0.2–1.0\right]$ ${\mathrm{mm}}^{-1}$ and $\mu_{a,1}=\mu_{a,2}-\Delta \mu_{a}$, $\Delta \mu_{a}^{\prime }=\left[0.008–0.05\right]$ ${\mathrm{mm}}^{-1}$. Expanding the boundaries of the selected ranges brought no benefits in the results, but increased the computation time. Figure 6b demonstrates the calculated intensities for particular sample parameters. It is clear that the region of uncertainty was much smaller for the liver tissue than for the gelatin-based phantom, and that it was below 0.5 mm. This allowed for freezing-depth sensing during a rather short cryo-application. In Figure 6c, the red circles indicate the estimated $\mu_{\mathrm{eff},\mathrm{th}}\left(t\right)$ for sample 4, demonstrating good agreement with the measured values.

The reconstructed freezing depth is shown in Figure 6d. We can see a rather different character of changing *L* for all the samples. This was associated with a number of factors. The main reason for such a strong variation was the different sizes of the liver lobes in the rats, which impacted on the heat transfer in their tissue. The differences of perfusion, initial optical properties and the state of a rat under anesthesia may also change the freezing rate. The observed results highlight the need for in situ monitoring of the damaged region. However, the final values of the estimated *L* are in good agreement with the visualized values. Note that due to the applied set-up, the measurement of *I* within a period of 3 s did not allow us to obtain signals exactly at 5 and 20 s. The results confirm the feasibility of the sapphire applicator to be applied for the sensing of freezing depth in vivo, despite the alteration of the sample properties.

## 4. Discussion

In this work, we demonstrate the concept of the sapphire cryo-applicator, which provides the ability to estimate tissue freezing depth using optical monitoring based on the steady-state analysis of diffuse reflected intensities. This allows for the monitoring of tissue freezing by means of rather simple optical equipment and three fiber channels in contrast to the previously proposed instrumentation that uses signal analysis in the frequency domain. The limits of possible depth reconstruction depend on the sample properties and separation between the applicator channels *R*. Our experimental approbation with tissue phantom demonstrated the upper limit of depth reconstruction, which is ∼8 mm, and the error of depth estimation < 1.5 mm. This error can be slightly improved by using the red or NIR light source and consequently increasing the penetration depth. It is also a significant drawback that it is difficult to estimate *L* at the beginning of sample freezing. However, the in vivo studies of the proposed applicator show the ability to reduce this blind area when scattering and absorption coefficients significantly increase. At the same time, from the theoretically estimated $I_{\mathrm{th}}\left(R,L\right)$, one can notice that this area depends on *R*. Thus, using smaller separation between the illumination and detection channels in the applicator, the bottom limit of the depth reconstruction can be also changed. It is clear that by increasing *R* one can broaden the upper limit for the *L* estimation. Therefore, adjusting the positions of the applicator’s channels, it is possible to choose the depth range that should be monitored. However, at high *L*, it is important to account for ice ball curvature. Thus, finding the trade-off between the depth and the reconstruction accuracy requires a separate study.

It is straightforward to roughly estimate the freezing depth using the behavior of $\mu_{\mathrm{eff}}$; in particular, its rate of change ${\dot{\mu }}_{\mathrm{eff}}$. From Figure 3e, we can see that it saturates with time. Therefore, the saturation of ${\dot{\mu }}_{\mathrm{eff}}$ can be applied as the marker of reaching a certain depth by the freezing front. At the same time, it is possible to involve the first derivative in the reconstruction procedure in order to increase the accuracy of Equation (8). We leave this for our further studies, in order to solve the task of improving *L* reconstruction.

Despite the cost of growing a sapphire crystal being higher than the cost of manufacturing a metal applicator, the higher freezing rate, the absence of tissue sticking to the contact surface and ability to withstand applications without surface degradation [37] make the usage of sapphire for cryosurgery cost-effective. We should highlight the use of the EFG technique for sapphire applicator manufacturing. It gives opportunities for choosing the appropriate crystal shape, changing the number of channels and their positions. It is important for transferring the proposed concept to clinical usage, assuming a significant variety of localizations and sizes of tissue lesions, as well as their possible heterogeneity. However, to study the performance of the sapphire applicator in these various conditions, experiments with more complex phantoms and tissue samples should be conducted.

The proposed concept of the sapphire applicator demonstrates its prospects for cryosurgery; however, it could find applications in other tasks, such as monitoring of tissue state and condition, for example, and detection of hematoma or tissue ishemia.

## 5. Conclusions

This work is aimed at solving the problem of monitoring tissue freezing depth during cryosurgery. For this, the concept of the sapphire cryo-applicator, which enables the analysis of a diffuse reflected optical response in a steady-state regime, is proposed. The advantages of this sapphire applicator are the minimal number of internal channels used for the illumination of a sample and the detection of optical radiation and the absence of frequency modulation of the light source. Our feasibility test of this applicator, made with a tissue-mimicking phantom, demonstrated its ability to sense tissue freezing depth up to 8 mm. The in vivo approbation of the sapphire applicator demonstrated the ability to detect the freezing depth despite the significant alteration of tissue properties. The results of this work, being a continuation of our previous studies, pave the way for further investigations of the sapphire cryo-applicator along with the development of other sapphire sensors for biophotonics and medicine.

## Data Availability

The data that support the findings of this study are available from the corresponding author upon reasonable request, due to privacy.
